# Acute fluoxetine exposure alters crab anxiety-like behaviour, but not aggressiveness

**DOI:** 10.1038/srep19850

**Published:** 2016-01-25

**Authors:** Trevor James Hamilton, Garfield T. Kwan, Joshua Gallup, Martin Tresguerres

**Affiliations:** 1 Department of Psychology, MacEwan University, Edmonton, AB, Canada, T5J 4S2.; 2 Neuroscience and Mental Health Institute, University of Alberta, Edmonton, AB, Canada, T6G 2H7.; 3 Marine Biology Research Division, Scripps Institution of Oceanography, University of California, San Diego, 9500 Gilman Drive, La Jolla, CA 92093, USA

## Abstract

Aggression and responsiveness to noxious stimuli are adaptable traits that are ubiquitous throughout the animal kingdom. Like vertebrate animals, some invertebrates have been shown to exhibit anxiety-like behaviour and altered levels of aggression that are modulated by the neurotransmitter serotonin. To investigate whether this influence of serotonin is conserved in crabs and whether these behaviours are sensitive to human antidepressant drugs; the striped shore crab, *Pachygrapsus crassipes*, was studied using anxiety (light/dark test) and aggression (mirror test) paradigms. Crabs were individually exposed to acute doses of the selective serotonin reuptake inhibitor, fluoxetine (5 or 25 mg/L), commonly known as Prozac®, followed by behavioural testing. The high dose of fluoxetine significantly decreased anxiety-like behaviour but had no impact on mobility or aggression. These results suggest that anxiety-like behaviour is more sensitive to modulation of serotonin than is aggressiveness in the shore crab.

The indolamine neurotransmitter serotonin (5-hydroxytryptamine; 5HT) plays a major role in the modulation of behaviour in vertebrate and invertebrate organisms^1^,^2^. The majority of serotonin receptors are G-protein coupled and activate intracellular signaling cascades that can cause long term changes in neuronal functioning resulting in alterations in behaviour^1^. Anxiety and aggression are two distinct behaviours that are complex in nature, rely on a variety of neurotransmitters and neuromodulators including serotonin, and are present throughout the animal kingdom. In the crab *Neohelice granulatus* (formerly *Chasmagnathus*), the level of aggressiveness is a major factor in the outcome of a fight and can be increased with release of serotonin^3^. Serotonin has also been shown to increase aggressive behaviour in lobsters^4^ and crayfish^5^, although this does not shift their dominance rank. However, other research has shown a decrease in aggressiveness after serotonin injection in crayfish^6^. Interestingly, the injection of serotonin can also increase anxiety-like behaviour in crayfish in a light/dark test, and this is reversed with the benzodiazepine anxiety reducing (anxiolytic) drug, chlordiazepoxide^7^. Therefore, evidence suggests that serotonin can alter aggression and anxiety in at least some invertebrate species but it is not known which of these behaviours is more sensitive to alterations in serotonin levels.

Fluoxetine, a selective serotonin reuptake inhibitor commonly known as Prozac®, causes a transient increase in serotonin levels at synapses throughout the nervous system. Additionally, fluoxetine has been reported to act as an anticonvulsant via the inhibition of persistent sodium channels^8^, an inhibitor of AMPA receptors^9^, and can also increase neurogenesis^10^. Fluoxetine is a popular pharmaceutical drug prescribed for the treatment of a variety of psychological disorders including depression, anxiety, obsessive-compulsive disorders, and nicotine addiction. As a result of its widespread use, there has been concern about its presence in wastewater effluents^11^,^12^. Environmentally relevant concentrations of antidepressants can alter reproduction, intracellular signalling pathways, memory, cognitive ability, activity, and development in some aquatic species^12^. Specifically, week-long fluoxetine exposure in shore crabs (*Carcinus maenas*) increased locomotion and increased the activity of muscle cholinesterases, glutathione *S*-transferases, glutathione reductase and total glutathiones^13^. This increased movement suggests that fluoxetine may act as an anxiolytic; however, specific tests were not conducted. In the current study we investigated whether acute fluoxetine exposure can alter anxiety and aggressiveness in the striped shore crab (*Pachygrapsus crassipes*).

## Results

### Open field test

**Figure 1:**
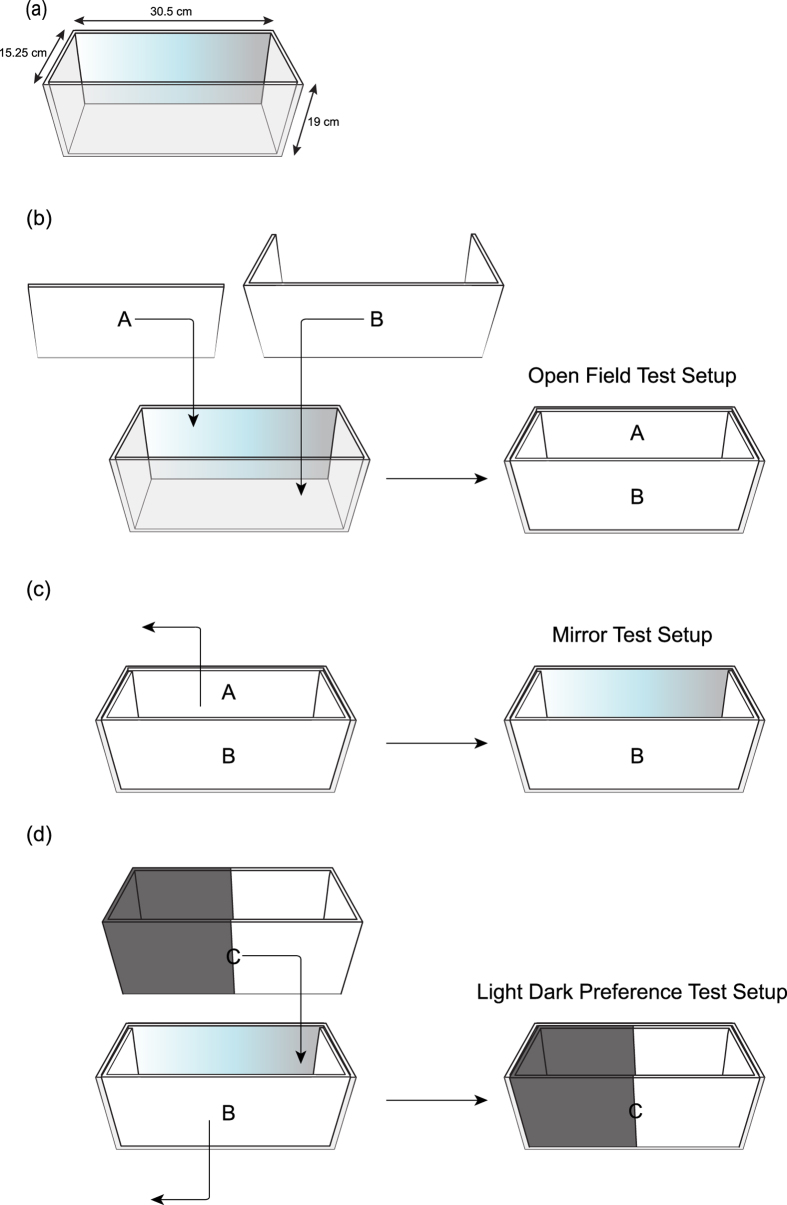
Diagrammatic representation of the aquaria (**a**) and panels (**A–C**) that were used to create the open field test (**b**), mirror approach test (**c**), and light/dark test (**d**).Blurred panels indicate the mirrors. Thin arrows indicate placement or removal of wall panels. Dimensions of zones used for quantification in Ethovision are also shown.

**Figure 2:**
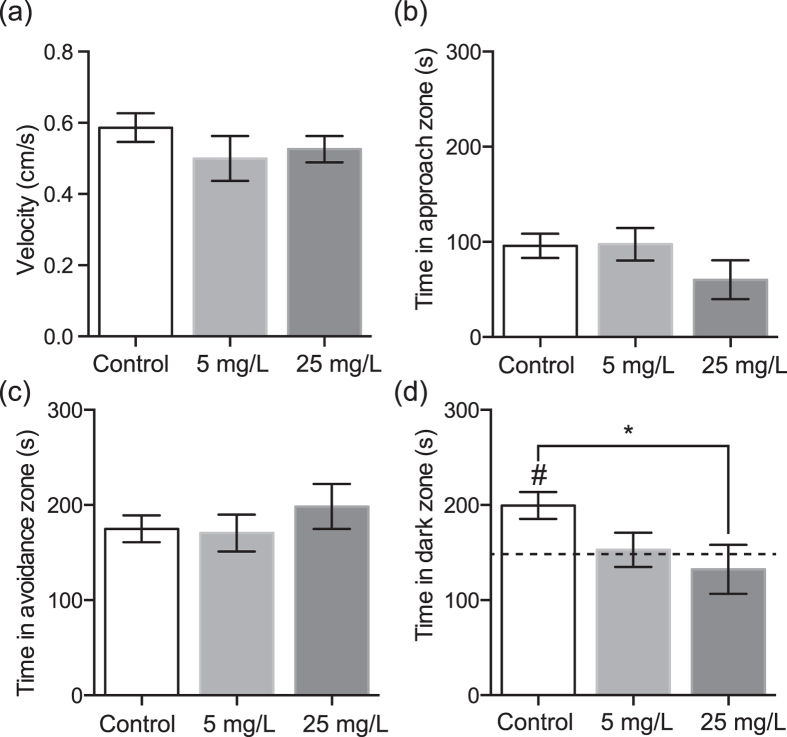
(**a**) Velocity during the open field test was not different between control and fluoxetine exposed groups (5 mg/L and 25 mg/L). (**b**) There were no significant differences between control and Fluox groups in the mirror-approach test for time in the approach zone, closest to the mirror, nor in the avoidance zone (**c**). (**d**) In the light/dark test only the control group had a significant dark preference (#, difference from 150, *p* = 0.0007). There was a significant difference between control and 25 mg/L Fluox groups (*p > 0.05).

After a 15-minute exposure to fluoxetine (5 or 25 mg/L) or control seawater, we found no difference in average velocity in the open-field test (Fig. 1A,B) between the control and fluoxetine groups (Fig. 2a; control: 0.59 ± 0.04 cm/s, fluoxetine 5 mg/L: 0.50 ± 0.06 cm/s; fluoxetine 25 mg/L: 0.53 ± 0.04 cm/s; Kruskal-Wallis test H (*N* = 107) = 4.855, p = 0.0883).

### Mirror approach test

We analyzed the time spent in all four corners of the mirror-approach arena (Fig. 1C) and found no significant difference between control (Control_ES1_: 170.7 ± 13.6 s) and fluoxetine 5 mg/L (160.8 ± 17.4; Mann-Whitney U test, *p* = 0.8568) and control (Control_ES2_: 224.6 ± 22.8) and fluoxetine 25 mg/L groups (161.0 ± 24.0; Mann-Whitney U test, *p* = 0.1511). We also analyzed the time spent in the avoidance zone, where the crabs were first positioned, and in the approach zone, closest to the mirror. We did not see a difference in the time spent in the approach zone (control: 95.8 ± 12.8 s, fluoxetine 5 mg/L: 97.5 ± 17.1 s; fluoxetine 25 mg/L: 60.2 ± 20.4 s; Kruskal-Wallis test H(N = 107) = 1.233, p = 0.5398; Fig. 2b) nor in the avoidance zone (control: 174.9 ± 14.2 s, fluoxetine 5 mg/L: 170.5 ± 19.4 s; fluoxetine 25 mg/L: 198.5 ± 23.5 s: Kruskal-Wallis test H(N = 107) = 1.134, p = 0.5673; Fig. 2c) between control and fluoxetine groups. Finally, we examined whether there was a difference in cornering preference for the mirror or non-mirrored side between groups. There was no difference in preference for cornering in the approach zone (next to the mirror) between the control (69.3 ± 11.2 s), fluoxetine 5mg/L (68.8 ± 11.8 s), or fluoxetine 25 mg/L (49.7 ± 19.1 s) groups (Kruskal-Wallis test H(N = 107) = 0.7187, p = 0.6981). There was also no difference in cornering preference for the non-mirrored side between the control (118.7 ± 13.9 s), fluoxetine 5mg/L (92.0 ± 18.1 s), and fluoxetine 25 mg/L (111.3 ± 24.4 s) groups (Kruskal-Wallis test H(N = 107) = 2.135, p = 0.3439).

### Light/dark test

Following the mirror approach test we administered the light/dark test (Fig. 1d). The control group had a significant preference for the dark zone (199.5 ± 13.9 s, p = 0.0007 Fig. 2d); however, exposure to fluoxetine abolished the dark preference (fluoxetine 5 mg/L: 152.9 ± 18 s, p = 0.778; fluoxetine 25 mg/L: 132.3 ± 25.7 s, p = 0.501); there also was a significant difference between groups (Kruskal-Wallis test: H (N = 107) = 6.414, p = 0.0405). A post-hoc Dunn’s multiple comparison test showed a significant difference between the control and fluoxetine 25 mg/L groups only (p < 0.05). There was no significant difference in velocity in the light/dark test between the three groups (control: 0.42 ± 0.04 cm/s, fluoxetine 5 mg/L: 0.55 ± 0.06 cm/s; fluoxetine 25 mg/L: 0.41 ± 0.03 cm/s; Kruskal-Wallis test H(N = 107) = 2.811, p = 0.2452).

## Discussion

This study suggests that the neuronal networks mediating anxiety-like behaviour in the shore crab *P. crassipes* are more sensitive to acute fluoxetine exposure than those controlling aggression. We found that exposure to 25 mg/L fluoxetine significantly reduced time in the dark zone (indicating reduced anxiety), but had no impact on approach to a mirror reflection or cornering (indicating no effect on aggression). Fluoxetine administration decreasing anxiety is consistent with similar studies in zebrafish with chronic exposure^14^. However, our findings that fluoxetine increases light preference are in direct contrast previous studies^7^,^15^, which found that serotonin injections increased dark preference in crayfish, and these were reversed with GABA_A_ agonist chlordiazepoxide and serotonin antagonists^7^,^15^. One explanation may be that increased exogenous serotonin via injection^7^,^15^ acts differently than increased endogenous serotonin caused by fluoxetine blocking the serotonin transporter(s). For example, the flood of serotonin produced by fluoxetine may cause increased autoreceptor activity that consequently decreases serotonin release. Fluoxetine injections had no effect on lobster aggressiveness^4^, consistent with our mirror-approach test findings. However, injections of fluoxetine together with serotonin had less of an effect than serotonin alone^4^, indicating that fluoxetine can act in opposition to the behavioural effects of serotonin injection. Thus, the action of endogenously released serotonin, which increases anxiety in crayfish^7^, could have been suppressed by fluoxetine, causing a decrease in anxiety in our study.

Chronic exposure to fluoxetine (eg. 750 ug/L over a week long period) can significantly increase locomotion in *C. maenas* crabs^13^. These results differ from the lack of change in average velocity shown here with an acute high dose of fluoxetine. There are a few potential explanations for this discrepancy including neuronal adaptation to chronic fluoxetine and species-specific differences in behaviour and response to fluoxetine, as well as the shape of the testing apparatus being a rectangle (here) compared to a circle^13^. With a circular arena the crab cannot engage in ‘cornering’ behaviour that has previously been related to dominant-subordinate classes in crayfish, with dominants cornering significantly more^16^. Thus, the presence of corners in our paradigm may have prevented crabs from moving around the arena. However, we did not find a significant difference in time spent in corners in our control vs. fluoxetine groups, so this is not likely the cause. A more likely and obvious explanation is the duration of exposure to fluoxetine, since in humans chronic exposure causes changes in serotinergic functioning that often takes weeks^17^. Taken together, the lack of difference in velocity across our treatment groups, but the abolishment of dark preference in fluoxetine-exposed crabs and significant difference in time spent in the dark zone for the controls compared to fluoxetine at 25 mg/L, indicates the drug altered their anxiety-like behaviour but not their locomotion.

The concentrations of fluoxetine used in the current study were many orders of magnitude larger than what has been reported in wastewater runoff^12^ and therefore, our results are not directly relevant to the environment. However, this study does suggest that the impact of wastewater effluent containing fluoxetine would first have an impact on neural circuitry mediating anxiety-like behaviour, over aggression or locomotion.

The involvement of serotonin in anxiety-like behaviour in crabs is consistent with vertebrates, including humans. The current study adds to the already large body of evidence indicating that this serotinergic pathway is ancient and evolutionarily conserved^1^,^18^.

## Methods

In this study, 109 *P. crassipes* crabs (mean weight ± S.E.M.: 3.03 ± 0.18 g; mean width ± S.E.M.: 1.91 ± 0.05 cm, 57 males and 52 females) were wild caught near Scripps Institution of Oceanography. Crabs were individually placed in a sequence of behavioural tests including the open-field test, mirror-aggression test, and light/dark test (see Fig. 1). Fluoxetine (Sigma, St Louis, MO, USA) was administered for 15 minutes at 5 mg/L or 25 mg/L prior to testing.

After being wild-caught, *P. crassipes* crabs were acclimated in large aquaria (180 × 76 × 85 cm) with aeration and flowing seawater (to a height of 35 cm) for 7–10 days prior to experimentation. Crabs were maintained on a 12:12 light dark cycle and fed a diet of frozen market squid (*Doryteuthis opalescens*) and pacific sanddab (*Citharichthys sordidus*), but were not fed on the experimental day. Experiments were performed in two experimental sets (ES) in June of 2014 (ES_1_) and June of 2015 (ES_2_) to account for any population differences. Molting has been observed annually in late fall in this species [personal observation^19^; and since molting hormones such as ecdysteroids can play a role in other biological processes in crabs^20^ and stress resistance in insects^21^ we chose a time period prior to the the molt cycle. Control groups were combined when there were no significant differences between ES_1_ and ES_2_. The only variable that had a significant difference between controls in ES_1_ and ES_2_ was for time spent in all of the corners of the mirror-approach arena.

### Behavioural Testing

Previous research on serotonin and aggression has primarily used tests that involve multiple crab or crayfish pairs or triads performing agonistic and antagonistic behaviours^3^,^4^,^16^. Since we focused on the effects of fluoxetine on two different behaviours, anxiety and aggression, we created a sequence of behavioural tests that can be performed without having to move the crab in and out of different testing apparatus. These procedures were designed to eliminate the handling of the crabs in between tests, in an attempt to minimize stress, an important factor because stressors are known to increase blood glucose and decrease dark preference in crayfish^7^. We modified a mirror response task previously used with crayfish^22^,^23^, hermit crabs^24^, and fiddler crabs^25^. Our experimental arena was designed so a crab could first habituate in an open-field test, then with the removal of one wall in the arena were exposed to the mirror (Fig. 1B,C). Prior to mirror exposure, crabs were gently moved to the center of the back wall of the arena (in the avoidance zone) then the mirror panel was removed, exposing the crabs to their reflection. Following this test, half light and half dark wall panels were inserted to create a light/dark test (Fig. 1c), known to measure anxiety-like behaviour in rodents^26^,^27^, many fish species^28^,^29^,^30^,^31^ and crayfish^7^. All testing trials were five minutes in duration, which is common in anxiety testing paradigms^14^,^32^,^33^,^34^ and were recorded and analyzed with Ethovision XT (v.10; Noldus Information Technology, Leesburg, VA, USA). Average velocity (cm/s) and time in zones (s) or corners (s) were quantified in Ethovision XT (see Fig. 1 for zone descriptions). One control experiment (ES_1_) and one fluoxetine experiment (ES_1_) were eliminated due to the crab crawling out of the arena.

### Drug Administration

Fluoxetine was purchased from Sigma (St Louis, MO) and was dissolved in reverse osmosis water (5 mg/ml) then applied in a 400 mL glass dosing container which had a plastic lid and was surrounded by a white barrier to block external stimuli. Before behavioural testing, crabs were individually immersed in the container with seawater (control crabs; ES_1_, n = 39; ES_2_, n = 18) or seawater containing fluoxetine (5 mg/L, ES_1_, n = 34, or 25 mg/L ES_2_, n = 18) for 15 minutes; this form of drug administration was chosen over injection in order to minimize the handling of the crabs. Crabs were randomly chosen and administered either fluoxetine or control treatments. To eliminate any potential effect of circadian cycles we tested during the day cycle and alternated the testing of control crabs and fluoxetine exposed crabs. Control groups for ES_1_ and ES_2_ had an equivalent amount of reverse osmosis water added to the dosing container.

### Statistical Methods

Normality was tested with the D’Agostino and Pearson omnibus test. Time in the dark zone was analyzed with one-sample *t*-tests or Wilcoxon signed-rank tests to assess statistically significant differences from 150 s. Two-tailed paired and unpaired *t*-tests and one-way ANOVAs were used for parametric data, and Mann–Whitney U-tests and Kruskal–Wallis tests with Dunn’s multiple comparison post hoc tests for data that were not normally distributed. An alpha-level of *P *< 0.05 and 95% confidence intervals were used for assessing statistical significance in all tests. Data were analysed using GraphPad PRISM v. 4.0B (La Jolla, CA, USA). Data are presented as mean ± s.e.m.

## Additional Information

**How to cite this article**: Hamilton, T. J. *et al*. Acute fluoxetine exposure alters crab anxiety-like behaviour, but not aggressiveness. *Sci. Rep.* **6**, 19850; doi: 10.1038/srep19850 (2016).
